# Age and sources of information variations and similarities on awareness of treatment and prevention of stroke among public and outpatients in Sub-Saharan Africa: a cross-sectional questionnaire study in Botswana

**DOI:** 10.1186/s12889-025-21900-7

**Published:** 2025-02-24

**Authors:** Ookeditse Ookeditse, Kebadiretse K. Ookeditse, Thusego R. Motswakadikgwa, Gosiame Masilo, Yaone Bogatsu, Baleufi C. Lekobe, Mosepele Mosepele, Henrik Schirmer, Stein H. Johnsen

**Affiliations:** 1 Department of Physical Medicine and Rehabilitation, Division of Neurorehabilitation Medicine, Trust Hospital in Vestfold, Kysthospitalet Sykehuset, Tønsberg, Norway; 2 Department of Internal Medicine, Division of Neurology and Neurorehabilitation Medicine, Sir Ketumile Masire Teaching Hospital, Gaborone, Botswana; 3 Department of Internal Medicine, Sidilega Private Hospital, Gaborone, Botswana; 4 Faculty of Medicine, University of Botswana, Gaborone, Botswana; 5 Department of Family Medicine and Occupational Medicine, Sidilega Private Hospital, Gaborone, Botswana; 6 Larvik Acute and Emergency Clinic, Larvik, Norway; 7 Division of Family Medicine, Nanset Medical Clinic, Larvik, Norway; 8 Princess Marina Referral Hospital, Gaborone, Botswana; 9 Institute of Clinical Medicine, UIT the Arctic University of Norway, Tromsø, Norway; 10 Department of Cardiology, Akershus University Hospital, Lørenskog, Norway; 11 Institute of Clinical Medicine, University of Oslo, Oslo, Norway; 12 Department of Neurology, University Hospital of North Norway, Tromsø, Norway

**Keywords:** Stroke prevention, Acute stroke treatment, Outpatients, Public, Awareness, Thrombolysis, Medical therapy

## Abstract

**Objectives:**

In this cross-sectional study from Botswana, we investigated awareness of acute stroke treatment and prevention among stroke-risk outpatients and the public based on age and sources of information, in addition to association of stroke risk factors with this awareness.

**Method:**

Questionnaires on awareness of acute stroke treatment and prevention were administered by research assistants to a representative selection of outpatients and the public.

**Results:**

The response rate was 93.0% for the public and 96.6% for outpatients. Public respondents had a mean age of 36.1 ± 14.5 years (age range 18–90 years) and 54.5% were females, while outpatients had a mean age of 37.4 ± 12.7 years (age range 18–80 years) and 58.1% were females. Awareness of medical therapy as acute stroke treatment was inadequate among outpatients (75.5% for public vs 43.4% for outpatients among all-age, *p* < 0.001), due to awareness differences among all ages. Awareness of stroke prevention was adequate (81.5% of outpatients vs 71.6% of public among all-age, *p* = 0.601%), and similar trend was observed also among individual age groups. For awareness of medical therapy as an acute stroke treatment among all ages, the public was more likely than outpatients to get stroke information (*p* < 0.001) from almost all sources of information, while for awareness of stroke prevention among all-age, outpatients were more likely than the public to get stroke information from family/ friends (83.9% vs 70.5%, *p* = 0.042).

History of HIV/AIDS and having a healthy diet were associated with lower awareness of both acute stroke treatment and prevention (*p* < 0.05).

**Conclusion:**

Results call for strategic educational stroke campaigns using best information relaying tool for each age and respondents’ group.

**Supplementary Information:**

The online version contains supplementary material available at 10.1186/s12889-025-21900-7.

## Highlights

• This is the first study comparing awareness of acute stroke treatment and prevention among outpatients and the public based on age and sources of information in sub-Saharan Africa

• Awareness of medical therapy as acute stroke treatment was lower among outpatients while higher among the public among all ages

• Almost all sources of stroke information were associated with higher awareness of acute stroke treatment among the public

• History of HIV/AIDS and having a healthy diet were associated with both poor awareness of acute stroke treatment and prevention

• Results call for educational stroke campaigns based on age and using almost all sources of information

**Supplementary Information:**

The online version contains supplementary material available at 10.1186/s12889-025-21900-7.

## Introduction

Stroke was the sixth most common cause of death in Africa in 2019, with an increasing trend from eight position in 2000, according to World Health Organization (WHO) estimates [1]. Globally, the highest age-standardized incidence of stroke is in Africa with the incidence of stroke increased in east Asia and southern sub-Saharan Africa (SSA) from 1990 to 2016 [2, 3].

The emergence of revascularization methods (intravenous thrombolysis and/or thrombectomy) as an effective treatment for acute ischemic stroke, have increased the focus on stroke as an urgent and emergency disease as these treatments are time-dependent, as they improve clinical outcomes and dependency in disability-adjusted life-years (DALYs) [4–8]. Some studies showed patients as the most predominant barrier in stroke care, (up to 91% as prehospital delay in thrombolytic therapy) while one systematic review study demonstrated very low rates of thrombolysis (19%) in low- and middle-income countries (LMIC) compared to at least 50% in high-income countries (HIC) [9–13].

Majority of studies have focused on knowledge of stroke symptoms and risk factors, while a few on awareness of treatment and prevention [14–25]. There is none that we know of that has focused on awareness based on both age and sources of information concurrently in Sub-Saharan Africa or worldwide.

## Objectives

1. To determine awareness of treatment and prevention of stroke among outpatients and the public based on age and sources of information.
2. To investigate if respondents’ sociodemographic and stroke risk factors are associated with stroke awareness.

## Methods

### Study design

Participants were recruited from Greater Gaborone which is located in the southeastern part of Botswana (is an upper middle-income country under LMIC in SSA), comprises six (6) districts and is the most populated area in the country.

### Sampling of study places and population

All names of the four (4) districts in Greater Gaborone except Gaborone City and Lobatse town were put in a box, and two (2) names were blindly selected from the box as study places in addition to Gaborone city and Lobatse town. This similar method was also applied for healthcare facilities and communities in the districts. For Gaborone city, all areas were categorized into three socio-economic groups i.e., low, middle and high income. Names of more than three (3) areas with similar socio-economic category were put in a box and three (3) names were blindly selected to represent that specific category. If there were only two areas in the same category, they were both included.

The study sampled a variety of two groups of respondents, namely the public with/without stroke risk factors and representing the general public, and medical outpatients with at least one stroke risk factor (history of hypertension, stroke, heart diseases, obesity, overweight, diabetes, and Human immunodeficiency virus (HIV/AIDS), smoking, family history of stroke/ heart diseases, sedentary lifestyle, psychiatric disorders, and unhealthy diet). Public respondents were recruited from their homes or workplaces in both rural and urban areas. Outpatients from both primary and secondary healthcare facilities while waiting for or after their medical consultation.

We did not calculate sample size since there are no studies with data on prevalence and/or incidence of stroke in Botswana that could have helped us to calculate it. Therefore, we aimed at recruiting at least 70% of those targeted during data collection period.

Trained research assistants interviewed respondents verbatim. Each interviewer conducted a standardized, structured, one-to-one interview, according to a multi-sectioned questionnaire designed to guide interview and avoid bias. For the public, no more than two respondents from the same family/compound/ company were interviewed. The interviewer only intervened when asked to clarify any question, without giving correct answers. We sampled only odd numbers for outpatients queuing at healthcare facilities and households for the public in each area. For the public, we further sampled from different socio-economic levels i.e., high, moderate and low socioeconomic areas within Greater Gaborone.

### Inclusion criteria

For the general public, respondents residing or working in Greater Gaborone, with or without any stroke risk factors. No more than two (2) respondents from same family, compound or company were included. Outpatients with at least one stroke risk factor visiting healthcare facilities in Greater Gaborone during study period but not admitted. Respondents aged ≥ 18 years. Respondents who understood English or local language, Setswana, and capable of consenting. Only respondents from the randomly selected places and not from the pilot study place were included.

### Exclusion criteria

Respondents with cognitive/speech difficulties.

### Data collection instrument

The survey instruments were adapted from previous surveys [14–25] with some modifications to reflect the recent American Heart Association/American Stroke Association (AHA/ASA) guidelines and European Stroke Organization guidelines [26, 27]. We tested the questionnaire in a pilot study with a sample of twenty-five respondents and some changes were made in the wording of questions based on the result of the pilot study. The questionnaire instruments were anonymous, electronic-based, written, and administered in English or local language (Setswana), consisting of both open- and closed-ended in nature and categorized into five sections (eFigure 1).

Sect. 1 included sociodemographic factors of respondents.

**Sociodemographic characteristics**: Variables included in this study were age (18–34 years, 35–49 years and ≥ 50 years), gender (male and female), education level (none/ unknown/ primary, secondary and tertiary), marital status (married/cohabiting and other), medical insurance (yes and no), and residing/ working together status (yes and no).

Sect. 2 comprised closed-ended questions on awareness of acute stroke treatment while Sect. 3 was made of both open- and closed-ended questions on awareness of stroke prevention.

**Awareness of acute stroke treatment**: It was assessed in 2 closed-ended questions. The first one: “Is acute stroke treatable?” Answers included yes, no, and no idea. The second question with 4 answers as follows: “Do you know any ways that can treat acute stroke?” Answers included no idea, rehabilitation, lifestyle, or medicine. We then dichotomized the responses into medicine vs other options.

**Awareness of stroke prevention**: It was assessed by both open- and closed-ended questions. “Is stroke preventable?” Answers included yes, no, or no idea. “Does reducing/treating stroke risk factors reduce likelihood of stroke?” Answers included no idea, no, or yes. We then dichotomized the responses into yes vs other options. “Do you know any ways that can reduce stroke risk factors?” The first part without answers and the second part with multiple choice answers: no idea, none, lifestyle, or medicine. Answers were then trichotomized into lifestyle vs medicine vs others.

Each correct answer in each Sect. 2 and 3 scored 1 point and was considered being aware. Each incorrect, unanswered, or unknown answer scored 0 point and was considered being unaware.

Section 4 and 5 comprised respondents’ stroke risk factors and sources of stroke information respectively.

### Respondents’ stroke risk factors

Refer to previous study [28].

Information on physical activity at work, at home, during recreational or sporting activities, and leisure-time activities was obtained using part of the International Physical Activity Questionnaire with comparable variables [29]. Participants were asked about the specific activities that they regularly perform that increase breathing rate for at least 10 min, total duration per day, number of days in a week, and whether the individual perceived the activity as heavy, moderate, light, or no activity. For each participant, recorded activities were converted to metabolic equivalent task (MET)-minutes per week (min/wk) [29]. People participating in activities of less than 3.5 MET-min/wk were classified as no activity (sedentary lifestyle), 3.5- < 600 MET-min/wk as low, 600- < 3000 MET-min/ wk as moderate, and ≥ 3000 MET-min/wk as high level of physical activity.

Respondents were asked if they perceived their weight as underweight, normal, overweight or obese. We further measured weight and height, calculated BMI, and classified as defined by the WHO and National Institutes of Health (NIH) i.e., underweight as BMI < 18·5 kg/m^2^, normal BMI 18.5- < 25 kg/m^2^, overweight 25- < 30 kg/m^2^, and obesity as ≥ 30 kg/m^2^ [30, 31]. Height was measured twice to the nearest millimeter using a fixed, non-elastic plastic stadiometer, and the average height calculated. Body weight was measured in kilograms (to the second decimal place) using an auto-zeroing digital weight scale for adults dressed in light clothes and without shoes. Safeway auto-zeroing digital weight scales (Safeway Scale, South Africa) were used after calibration.

### Sources of stroke information

Refer to previous study [28]. Sources of information were then stratified by awareness of acute stroke treatment and prevention.

### Statistical analysis

Continuous variables were expressed as mean ± standard deviation (SD) or 95% confidence interval (CI). Categorical and ordinal variables were expressed as absolute frequency (n) and proportion (%) of the overall sample or subgroups. Outpatients and public groups’ awareness of stroke stratified by age and sources of information was compared using the chi-square test. Odds ratios were used to analyze awareness of both acute stroke treatment and prevention [32].

Mann–Whitney U/ Kruskal–Wallis H was used to assess association of respondents’ sociodemographic and stroke risk factors with awareness of stroke prevention and treatment. Bonferroni correction was applied for multiple comparisons. Statistical tests were two-tailed and reported statistically significant at *p* < 0.05. All statistical analyses were completed using SPSS 29 statistical software (SPSS Inc., Chicago, Illinois, USA).

## Results

We interviewed 2987 respondents in a cross-sectional study from June-October 2019. We excluded 179 participants (151 from the public and 28 outpatients) because of missing either consent or substantial information that could be useful for the study (eFigure 2). We had a valid response of 2808 respondents (94.0%), comprising 2013 from the public (93.0%) and 795 outpatients (96.6%). The public had a mean age of 36.1 ± 14.5 years (age range 18–90 years), while outpatients had 37.4 ± 12.7 years (range 18–80 years). Public and outpatients comprised 54.8% and 57.2% females respectively. More information on respondents’ characteristics is elucidated in Table 1.

**Table 1:** Sociodemographic and stroke risk factors among respondents

	Total	Public	Outpatients	
	n = 2808	n = 2013	n = 795	
	n (%)	n (%)	n (%)	***p***
**Sociodemographic factors**				
**Gender**				
Female	1559(55.5)	1104(54.8)	455(57.2)	
Male	1249(44.5)	909(45.2)	340(42.8)	0.251
**Age category (years)**	missing 6	missing 5	missing 1	
18–34	1501(53.6)	1118(55.7)	383(48.2)	
35–49	842(30.0)	586(29.2)	256(32.2)	
≥50	459(16.4)	304(15.1)	155(19.5)	< 0.001
**Education level**				
Primary or below	363(12.9)	257(12.8)	106(13.3)	
Secondary	1518(54.1)	1108(55.0)	410(51.6)	
Tertiary	923(32.9)	645(32.0)	278(35.0)	
Unknown	4(0.1)	3(0.1)	1(0.1)	0.405
**Medical insurance**				
Yes	420(15.0)	92(4.6)	328(41.3)	
No	2388(85.0)	1921(95.4)	467(58.7)	< 0.001
**Marital status**				
Married/cohabiting	982(35.0)	723(35.9)	259(32.6)	
Others	1826(65.0)	1290(64.1)	536(67.4)	0.095
**Residing/working together**				
Yes	1121(39.9)	954(47.4)	167(21;0)	
No	1687(60.1)	1059(52.6)	628(79.0)	< 0.001
**Self-reported risk and other factors**				
**History of hypertension**				
Yes	276(9.8)	158(7.8)	118(14.8)	
No	2532(90.2)	1855(92.2)	677(85.2)	< 0.001
**History of CVDS**				
Yes	196(7.0)	116(5.8)	80(10.1)	
No	2612(93.0)	1897(94.2)	715(89.9)	< 0.001
**Family history of stroke/heart diseases**				
Stroke	372(13.2)	285(14.2)	87(10.9)	
Heart diseases	347(12.4)	221(11.0)	126(15.8)	
Both heart diseases and stroke	389(13.9)	202(10.0)	187(23.5)	
None	1700(60.5)	1305(64.8)	395(49.7)	< 0.001
**BMI**				
Underweight	53(1.9)	31(1.5)	22(2.8)	
Normal	2429(86.5)	1850(91.9)	579(72.8)	
Overweight	215(7.7)	120(6.0)	95(11.9)	
Obesity	111(4.0)	12(0.6)	99(12.5)	< 0.001
**Healthy dietary status**				
No	1080(38.5)	772(38.4)	308(38.7)	
Yes	1691(60.2)	1213(60.3)	478(60.1)	
Unknown	37(1.3)	28(1.4)	9(1.1)	0.855
**Alcohol consumption**				
Current	668(23.8)	415(20.6)	253(31.8)	
Former	46(1.6)	25(1.2)	21(2.6)	
No	2086(74.3)	1569(77.9)	517(65.0)	
Unknown	8(0.3)	4(0.2)	4(0.5)	< 0.001
**Smoking status**				
Current	337(12.0)	198(9.8)	139(17.5)	
Former	43(1.5)	22(1.1)	21(2.6)	
No	2421(86.2)	1788(88.8)	633(79.6)	
Unknown	7(0.2)	5(0.2)	2(0.3)	< 0.001
**Intensity of physical activity**				
None	2157(76.8)	1573(78.1)	584(73.5)	
Light	105(3.7)	62(3.1)	43(5.4)	
Moderate	483(17.2)	337(17.0)	140(17.6)	
High	63(2.2)	35(1.7)	28(3.5)	< 0.001
**History of HIV/AIDS**				
Yes	567(20.3)	328(16.3)	239(30.1)	
No	2241(79.8)	1685(83.7)	556(69.9)	< 0.001
**History of psychiatric diseases**				
Yes	89(3.2)	0(0)	89(11.2)	
No	2719(96.8)	2013(100.0)	706(88.8)	< 0.001
**Calculated risk factors**				
**Physical activity intensity (Met min/week)**				
Physical inactive	2169(77.2)	1575(78.2)	594(74.7)	
Low (> 3,5–600)	112(4.0)	75(3.7)	37(4.7)	
Moderate (> 600–3000)	436(15.5)	294(14.6)	142(17.9)	
High (> 3000)	91(3.2)	69(3.4)	22(2.8)	0.078
**BMI**				
Underweight (< 18.5)	105(3.7)	77(3.8)	28(3.5)	
Normal (18.5 < 25)	1169(41.6)	877(43.6)	292(36.7)	
Overweight (25 < 30)	669(23.8)	475(23.6)	194(24.4)	
Obesity (≥ 30)	856(30.5)	579(28.8)	277(34.8)	
Unknown	9(0.3)	5(0.2)	4(0.5)	0.005

*NA *not applicable, *CVDS *cardiovascular diseases (diabetes, dyslipidemia, or heart diseases)

Psychiatric diseases: depression or anxiety, *BMI *Body Mass Index, P:calculated using chi-squared

### Awareness of acute stroke treatment

About 9 out of 10 respondents (88.7%) were aware of acute stroke treatment (90.8% of outpatients vs 87.9% of public), while 66.4% recognized medical therapy as acute stroke treatment (75.5% of public vs 43.3% of outpatients, *p* < 0.001) (Table 2). For all respondents, the odds of being aware of both acute stroke treatment and medical therapy as a way of acute treatment was 1873 times (for public 2949 vs for outpatients 133 times) higher than being aware of acute stroke treatment only (*p* < 0.001) (eTable 1).

**Table 2:** Public and outpatients awareness of acute treatment and prevention of stroke stratified by age

		Total	Outpatients	Public	
	Age (years)	n(%)	n(%)	n(%)	*p*
**Awareness of acute stroke treatment**					
	All	2492(88.7)	721(90.8)	1766(87.9)	0.606
	18–34	1323(88.1)	345(90.1)	978(87.5)	0.742
	35–49	757(89.9)	232(90.6)	525(89.6)	0.920
	> 50	407(88.7)	144(92.9)	263(86.5)	0.627
**Awareness of medical therapy as acute stroke treatment**					
	All	1864(66.4)	344(43.3)	1517(75.5)	< 0.001
	18–34	1016(67.7)	168(43.9)	848(75.8)	< 0.001
	35–49	549(65.2)	106(41.4)	443(75.6)	< 0.001
	> 50	296(64.5)	70(45.2)	226(74.3)	0.006
**Awareness of stroke prevention**					
	All	2089(74.4)	647(81.5)	1437(71.6)	0.055
	18–34	1124(74.9)	305(79.6)	819(73.3)	0.383
	35–49	619(73.5)	208(81.3)	411(70.1)	0.228
	> 50	341(74.3)	134(86.5)	207(68.1)	0.135
**Awareness of stroke risk factor prevention as stroke prevention**					
	All	2471(88.0)	751(94.6)	1714(85.4)	0.102
	18–34	1334(88.9)	363(94.8)	971(86.9)	0.321
	35–49	737(87.5)	242(94.5)	495(84.5)	0.316
	> 50	394(85.8)	146(94.2)	248(81.6)	0.337
**Spontaneously recalling at least one way of preventing stroke risk factors**					
	All	932(33.2)	246(31.0)	686(34.2)	0.353
	18–34	531(35.4)	133(34.7)	398(35.6)	0.860
	35–49	288(34.2)	76(29.7)	212(36.2)	0.284
	> 50	113(24.6)	37(23.9)	76(25.0)	0.865
**Identifying lifestyle changes/medicine as a way of prevention of stroke risk factors**					
	All	2648(94.3)	765(96.3)	1877(93.5)	0.620
	18–34	1414(94.2)	365(95.3)	1049(93.8)	0.857
	35–49	810(96.2)	252(98.4)	558(95.2)	0.759
	> 50	418(91.1)	148(95.5)	270(88.8)	0.621
**Identified none of the ways of preventing stroke risk factors**					
	All	160(5.7)	29(3.7)	131(6.5)	0.031
	18–34	87(5.8)	18(4.7)	69(6.2)	0.450
	35–49	32(3.8)	4(1.6)	28(4.8)	0.082
	> 50	41(8.9)	7(4.5)	34(11.2)	0.084
**Spontaneously recalled ways of preventing stroke risk factors**					
**Physical activity**					
	All	661(23.5)	183(23.0)	478(23.8)	0.800
	18–34	384(25.6)	102(26.6)	282(25.2)	0.742
	35–49	197(23.4)	55(21.5)	142(24.2)	0.587
	> 50	80(17.4)	26(16.8)	54(17.8)	0.867
**Healthy diet**					
	All	358(12.8)	100(12.6)	258(12.8)	0.910
	18–34	218(14.5)	56(14.6)	162(14.5)	0.965
	35–49	99(11.8)	28(10.9)	71(12.1)	0.743
	> 50	41(8.9)	16(10.3)	25(8.2)	0.613
**Avoid/reduce high alcohol intake**					
	All	132(4.7)	36(4.5)	96(4.8)	0.851
	18–34	82(5.5)	23(6.0)	59(5.3)	0.711
	35–49	36(4.3)	11(4.3)	25(4.3)	0.980
	> 50	14(3.1)	2(1.3)	12(3.9)	0.232
**Cessation of smoking**					
	All	106(3.8)	31(3.9)	75(3.7)	0.881
	18–34	63(4.2)	17(4.4)	46(4.1)	0.855
	35–49	33(3.9)	12(4.7)	21(3.6)	0.602
	> 50	10(2.2)	2(1.3)	8(2.6)	0.481
**Avoid stress**					
	All	69(2.5)	18(2.3)	51(2.5)	0.769
	18–34	37(2.5)	10(2.6)	27(2.4)	0.874
	35–49	27(3.2)	6(2.3)	21(3.6)	0.496
	> 50	5(1.1)	2(1.3)	3(1.0)	0.844
**Medication adherence/follow-ups/education from healthcare professionals**					
	All	59(2.1)	20(2.5)	39(1.9)	0.512
	18–34	32(2.1)	10(2.6)	22(2.0)	0.618
	35–49	21(2.5)	7(2.7)	14(2.4)	0.843
	> 50	6(1.3)	3(1.9)	3(1.0)	0.558
**Reducing fatty foods intake**					
	All	57(2.0)	12(1.5)	45(2.2)	0.369
	18–34	32(2.1)	6(1.6)	26(2.3)	0.508
	35–49	19(2.3)	5(2.0)	14(2.4)	0.774
	> 50	6(1.3)	1(0.6)	5(1.6)	0.505
**Reducing salt consumption**					
	All	33(1.2)	10(1.3)	23(1.1)	0.859
	18–34	15(1.0)	4(1.0)	11(1.0)	0.934
	35–49	11(1.3)	4(1.6)	7(1.2)	0.751
	> 50	7(1.5)	2(1.3)	5(1.6)	0.818
**Eating more fruits and vegetables**					
	All	15(0.5)	1(0.1)	14(0.7)	0.119
	18–34	12(0.8)	0(0.0)	12(1.1)	0.059
	35–49	3(0.4)	1(0.4)	2(0.3)	0.930
	> 50	0(0.0)	0(0.0)	0(0.0)	NA
**Lose weight**					
	All	7(0.2)	2(0.3)	5(0.2)	0.991
	18–34	5(0.3)	2(0.5)	3(0.3)	0.638
	35–49	1(0.1)	0(0.0)	1(0.2)	0.552
	> 50	1(0.2)	0(0.0)	1(0.3)	0.552

*NA *not applicable, *p*: calculated using chi-squared

### Awareness of stroke prevention

A total of 2089 respondents (74.4%) were aware of stroke prevention (81.5% of outpatients vs 71.6% of public), 88.0% were aware that stroke risk factors’ prevention (reducing/ treating stroke risk factors) reduced the likelihood of stroke (94.6% outpatients vs 85.4% public), while 33.2% recalled spontaneously at least one way of preventing stroke risk factors (31.0% of outpatients vs 34.2% of public) (Table 2).

About 9 out of 10 outpatients (96.3%) and 93.5% of public identified either lifestyle changes or medicine/medication as a way of preventing stroke risk factors, while 3.7% of outpatients vs 6.5% of public (*p* < 0.05) could not identify any means of preventing stroke risk factors.

About 1 out of 4 respondents (23.5%) spontaneously recalled physical activity which was the highest way recalled (23.8% of public vs 23.0% of outpatients), while 0.2% recalled losing weight to maintain normal weight as lowest (0.3% of outpatients vs 0.2% of public) way of preventing stroke risk factors (Table 2).

For all respondents, odds of being aware of both prevention of stroke and reducing or treating stroke risk factors was 21 times (for public 22 vs for outpatients 14 times) higher than of being aware of stroke prevention only (*p* < 0.001) (eTable 1).

### Sources of stroke information

**Fig. 1:**
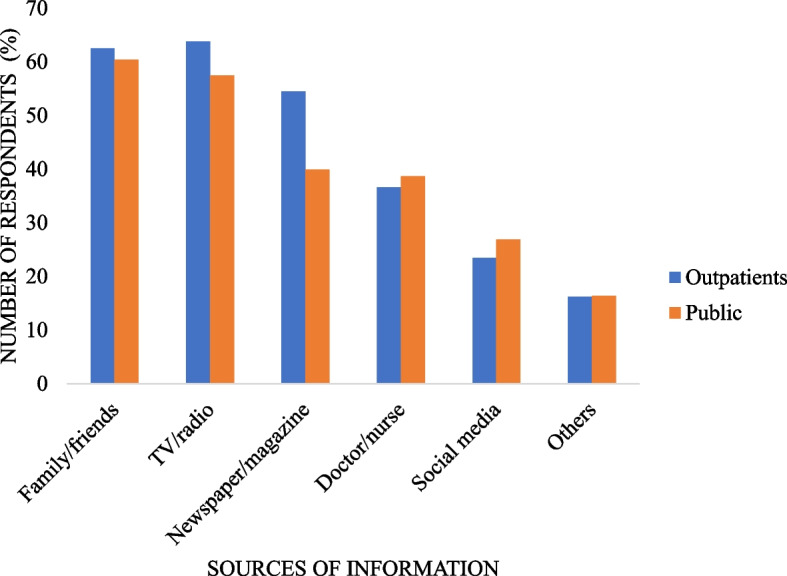
Sources of stroke information among the public and outpatients

Highest sources of information were TV/radio (63.8% for outpatients vs 57.5% for public) and family/friends (62.5% for outpatients vs 60.4% for public) while the lowest was others category (16.2% for outpatients vs 16.4% for public)) (Fig. 1). Outpatients were more likely than the public to get stroke information from newspaper/magazine (54.5% vs 39.9%, *p* < 0.001).

Regarding medical therapy as acute stroke treatment among almost all ages, the public was more likely than outpatients to get stroke information (*p* < 0.001) from almost all sources of information (Table 3). For stroke prevention, both groups demonstrated awareness rates of at least 67% for almost all sources except for others category among the public (63.0%). Outpatients were more likely than the public to get stroke information from family/ friends (83.9% vs 70.5%, *p* < 0.05).

**Table 3:** Respondents awareness differences of acute stroke treatment and prevention by sources of stroke information stratified by age

	**Public**		**Outpatients**		
Age(years)	n = 2008	No. aware (% aware)	n = 794	No. aware (% aware)	*p*
**Medical therapy as acute treatment**					
**Family or friends**					
All	1216	938(77.1)	496	238(48.0)	< 0.001
18–34	651	497(76.3)	241	121(50.2)	0.002
35–49	370	292(78.9)	147	64(43.5)	< 0.001
≥50	195	149(76.4)	108	53(49.1)	0.041
**Tv or radio**					
All	1152	953(82.5)	506	190(37.5)	< 0.001
18–34	650	543(83.5)	228	84(36.8)	< 0.001
35–49	337	276(81.9)	179	66(36.9)	< 0.001
≥50	165	131(79.4)	99	40(40.4)	0.005
**Newspaper or magazine**					
All	799	675(84.5)	432	162(37.5)	< 0.001
18–34	463	407(87.9)	204	81(39.7)	< 0.001
35–49	256	209(81.6)	154	52(33.8)	< 0.001
≥50	80	59(73.8)	74	29(39.2)	0.041
**Social Media (Internet, Facebook, WhatsApp)**					
All	542	456(84.1)	187	59(31.7)	< 0.001
18–34	312	269(86.2)	109	40(36.7)	< 0.001
35–49	194	159(82.0)	57	14(24.6)	< 0.001
≥50	36	28(77.8)	20	5(25.0)	0.055
**Doctor or nurse**					
All	777	674(86.7)	290	112(38.6)	< 0.001
18–34	438	399(91.1)	139	58(41.7)	< 0.001
35–49	245	201(82.0)	97	32(33.0)	< 0.001
≥50	94	74(78.7)	54	22(40.7)	0.040
**Others (school, patients, experience)**					
All	330	185(56.1)	129	74(57.4)	0.906
18–34	208	122(58.7)	83	48(57.8)	0.952
35–49	78	42(53.8)	22	14(63.6)	0.705
≥50	44	21(47.7)	24	12(50.0)	0.918
**Stroke prevention**					
**Family or friends**					
All	1216	857(70.5)	496	416(83.9)	0.042
18–34	651	476(73.1)	241	200(83.0)	0.293
35–49	370	250(67.6)	147	122(83.0)	0.198
≥50	195	131(67.2)	108	94(87.0)	0.182
**Tv or radio**					
All	1152	896(77.8)	506	435(86.0)	0.228
18–34	650	526(80.9)	228	189(82.9)	0.843
35–49	337	248(73.6)	179	155(86.6)	0.266
≥50	165	122(73.9)	99	91(91.9)	0.273
**Newspaper or magazine**					
All	799	645(80.7)	432	388(89.8)	0.241
18–34	463	393(84.9)	204	176(86.3)	0.898
35–49	256	187(73.0)	154	142(92.2)	0.144
≥50	80	65(81.3)	74	70(94.6)	0.535
**Social Media (Internet, Facebook, WhatsApp)**					
All	542	420(77.5)	186	160(86.0)	0.427
18–34	312	256(82.1)	109	93(85.2)	0.823
35–49	194	138(71.1)	57	49(86.0)	0.434
≥50	36	26(72.2)	20	18(90.0)	0.614
**Doctor or nurse**					
All	777	608(78.2)	290	246(84.8)	0.454
18–34	438	367(83.8)	139	116(83.5)	0.976
35–49	245	173(70.6)	97	84(86.6)	0.288
≥50	94	68(72.3)	54	46(85.2)	0.549
**Others (school, patients, experience)**					
All	330	208(63.0)	129	99(76.7)	0.263
18–34	208	127(61.1)	83	68(81.9)	0.177
35–49	78	58(74.4)	22	17(77.3)	0.922
≥50	44	23(52.3)	24	14(58.3)	0.828

*p*: calculated using chi-squared

### Respondents’ awareness differences of medical therapy as acute stroke treatment, and prevention by sources of stroke information stratified by age

Odds of being aware of medical therapy as acute stroke treatment due to getting information from family/friends were 1.7 times higher for outpatients while 1.2 times higher for the public than being aware without getting information from this source (eTable 2). Odds were also at least 2 times higher for the public due to getting information from doctor/nurse, newspaper/magazine, TV/radio, and social media while for outpatients, odds were 0.7 times or less lower due to getting information from these sources.

Odds of being aware of stroke prevention due to getting information from TV/radio and newspaper/magazine were at least 2 times higher (2 times for the public vs 2.2 times higher for outpatients, and 2.2 times for the public vs 3.5 times higher for outpatients respectively) than being aware without getting information from these sources (eTable 2).

Odds were also more than 1 time higher for outpatients due to getting information from family/friends while for the public due to getting information from doctor/nurse and social media.

### Association of sociodemographic factors with awareness of acute stroke treatment (medical therapy) and prevention

Lower awareness of acute stroke treatment (*p* < 0.05) was associated with medical insurance, not residing/working together, history of hypertension, history of CVDS, family history of both stroke and heart diseases, healthy diet, current drinkers, former drinkers, current smokers, history of HIV/AIDS, light and moderate physical intensity (Table 4). Lower awareness of stroke prevention (*p* < 0.05) was associated with female gender, no medical insurance, married/cohabiting status, residing/ working together, family history of stroke, healthy diet, history of HIV/AIDS, history of psychiatric diseases, underweight, normal weight, and overweight.

**Table 4:** Mann–Whitney U/ Kruskal–Wallis H- Association of awareness of acute stroke treatment and prevention with sociodemographic factors among respondents

	**Medical therapy as acute treatment**	**Prevention**
	Mean (95% CI)	Mean (95% CI)
**Sociodemographic factors**		
**Gender**		
Female	0.66(0.64–0.68)	0.73(0.71–0.75)
Male	0.67(0.64–0.69)	0.76(0.74–0.79)
*p*	0.636	0.038
*d*		0.039
**Age category (years)**		
1. 18–34	0.68(0.65–0.70)	0.75(0.73–0.77)
2. 35–49	0.65(0.62–0.68)	0.74(0.71–0.77)
3. ≥ 50	0.64(0.60–0.69)	0.74(0.70–0.78)
*p*	0.300	0.767
*d*		
**Education level**		
1.Primary, or unknown	0.68(0.63–0.73)	0.73(0.68–0.78)
2. Secondary	0.67(0.64–0.69)	0.74(0.71–0.76)
3. Tertiary	0.65(0.62–0.68)	0.76(0.73–0.79)
*p*	0.497	0.313
*d*		
**Medical insurance**		
Yes	0.44(0.40–0.49)	0.83(0.79–0.86)
No, unknown	0.70(0.68–0.72)	0.73(0.71–0.75)
*p*	< 0.001	< 0.001
*d*	0.196	0.079
**Marital status**		
Married/ cohabiting	0.67(0.64–0.70)	0.71(0.69–0.74)
Others	0.66(0.64–0.68)	0.76(0.74–0.78)
*p*	0.550	0.010
*d*		0.049
**Residing/working together**		
Yes	0.74(0.71–0.76)	0.70(0.67–0.72)
No	0.61(0.59–0.64)	0.77(0.75–0.79)
*p*	< 0.001	< 0.001
*d*	0.128	0.087
**Self-reported risk factors**		
**History of hypertension**		
Yes	0.61(0.55–0.66)	0.74(0.69–0.79)
No, unknown	0.67(0.65–0.69)	0.74(0.73–0.76)
*p*	0.030	0.847
*d*	0.041	
**History of CVDS**		
Yes	0.59(0.52–0.66)	0.77(0.71–0.83)
No	0.67(0.65–0.69)	0.74(0.73–0.76)
*p*	0.027	0.478
*d*	0.042	
**Family history of stroke/heart diseases**		
1. None	0.69(0.67–0.71)	0.76(0.74–0.78)
2. Both heart diseases and stroke	0.58(0.53–0.63)	0.79(0.75–0.83)
3. Heart diseases	0.63(0.58–0.68)	0.71(0.66–0.76)
4. Stroke	0.67(0.62–0.72)	0.66(0.61–0.71)
*p*	< 0.001; 1 vs 2 < 0.001	< 0.001; 1 vs 4 < 0.001, 2 vs 4 < 0.001
*d*	0.006	0.008
**Healthy dietary status**		
No, unknown	0.69(0.66–0.72)	0.78(0.76–0.80)
Yes	0.65(0.62–0.67)	0.72(0.70–0.74)
*p*	0.016	< 0.001
*d*	0.045	0.067
**Alcohol consumption**		
1. No, unknown	0.69(0.67–0.71)	0.74(0.72–0.76)
2. Current	0.59(0.55–0.62)	0.76(0.73–0.79)
3. Former	0.50(0.35–0.65)	0.76(0.63–0.89)
*p*	< 0.001; 1 vs 3 = 0.019, 1 vs 2 < 0.001	0.502
*d*	0.011	
**Smoking status**		
1. No, unknown	0.68(0.66–0.70)	0.75(0.73–0.77)
2. Current	0.54(0.49–0.60)	0.71(0.66–0.76)
3. Former	0.60(0.45–0.76)	0.74(0.61–0.88)
*p*	< 0.001; 1 vs 2 < 0.001	0.362
*d*	0.009	
**History of HIV/AIDS**		
Yes	0.50(0.45–0.54)	0.64(0.60–0.68)
No, unknown	0.71(0.69–0.73)	0.77(0.75–0.79)
*p*	< 0.001	< 0.001
*d*	0.179	0.115
**History of psychiatric diseases**		
Yes	0.75(0.66–0.84)	0.65(0.55–0.75)
No	0.66(0.64–0.68)	0.75(0.73–0.76)
*p*	0.071	0.043
*d*		0.038
**Calculated risk factors**		
**Intensity of physical activity**		
1. None, unknown	0.69(0.67–0.70)	0.75(0.73–0.77)
2. Light	0.56(0.47–0.66)	0.67(0.58–0.76)
3. Moderate	0.59(0.55–0.64)	0.73(0.69–0.77)
4. High	0.63(0.53–0.73)	0.78(0.69–0.87)
*p*	< 0.001; 1 vs 2 = 0.044, 1 vs 3 = 0.001	0.180
*d*	0.007	
**BMI**		
1. Underweight (< 18.5)	0.65(0.55–0.74)	0.62(0.52–0.71)
2. Normal, unknown (18.5 < 25)	0.65(0.62–0.67)	0.71(0.69–0.74)
3. Overweight (25 < 30)	0.66(0.62–0.69)	0.74(0.70–0.77)
4. Obesity (≥ 30)	0.70(0.67–0.73)	0.81(0.78–0.83)
*p*	0.111	< 0.001; 1 vs 4 < 0.001, 2 vs 4 < 0.001, 3 vs 4 = 0.010
*d*		0.011

*NA *not applicable, *CVDS *cardiovascular risk factors (diabetes, dyslipidemia, stroke, or heart diseases), d: effect size

Psychiatric diseases: depression or anxiety, BMI: Body Mass Index, MI: myocardial infarction

## Discussion

In this study, awareness of acute stroke treatment was adequate among respondents, with both the public and outpatients scoring more than 85% among all ages and without any significant differences. Despite this, only 3 out of 5 respondents (66.4%) recognized medical therapy as a way of acute stroke treatment i.e., 43.4% of outpatients vs 75.5% of public for all-age due to similar trend for all ages. These rates are lower compared to a Canadian study by Metias et al. comparing 2 cohorts (2010 and 2015), that showed public and patients had higher awareness rates of 87.9% in 2010 and 82.5% in 2015 [17]. Our findings for outpatients however, demonstrated higher awareness than in some patients’ studies [15, 16, 18–20] that showed rates between 3.6–26.2%. It is uncertain why the public had more awareness than outpatients when one would have expected outpatients to have better awareness since they are more frequently in contact with healthcare professionals. The reasons could be due to that outpatients are already preoccupied with their diseases as only few of our outpatients had stroke in this study, while the public have probably more time to learn about various diseases. The other reason could be that the public get stroke information from other sources which outpatients do not use or have access to.

We found that awareness of stroke risk factors’ prevention (closed-ended question) was adequate for outpatients and the public among all ages (lowest score about 80%) while awareness in an open-ended question was inadequate for both respondents among all ages (highest score less than 37%). In both groups, 81 −95% identified at least one way of preventing stroke risk factors while only one third spontaneously recalled at least one. Even though awareness of stroke prevention was sufficient, there was low awareness of spontaneously recalling means of preventing stroke risk factors. Some previous patients’ studies [21–25] have shown variations in awareness rates (21.7–90.8%).

Physical activity (regular exercises) was spontaneously recalled by 23.0% of outpatients, reducing/avoiding excessive alcohol drinking by 4.5%, quitting smoking by 3.9%, reducing fatty foods intake by 1.5%, whereas only 0.1% recalled eating more fruits/ vegetables. In an Ethiopian closed-ended study among hypertensive patients [21], the corresponding numbers were 27.1%, 62.7%, 51.7%, 24.1%. and 7.8%. Also, the awareness of medication adherence/treating or controlling cardiovascular diseases (2.5%) and good weight control (0.3%) were much lower in our study compared to the Ethiopian study [21]. Although some of the differences could be attributed to different study population (mixture of various cardiovascular diseases vs. hypertensives), the low numbers clearly indicate a great need for improved public health and lifestyle information in Botswana.

Among all ages, the public had significantly higher awareness than outpatients for medical therapy as acute stroke treatment among those who got stroke information from all sources i.e., doctors/ nurses, social media, newspapers/magazines, TV/ radio, and family/ friends.

Outpatients’ awareness by different sources of information ranged between 24.6% and 63.6%, which is insufficient, compared to the public who had the lowest awareness rates of 47.7–58.7% for others category, while others were more than 73%. This demonstrates that all sources of information can be used effectively to relay stroke information as shown among the public. This calls for more effort and using the best tools for each age group among outpatients to effectively educate them about stroke if we are to reduce stroke morbidities and mortalities. Awareness rates of stroke prevention by sources of information were high, with outpatients among all-age having a higher rate than the public among those who got stroke information from family/ friends. Despite this, the public among all-age had awareness rate of 70.5% which is sufficient. This could be due to that outpatients listen to and trust mostly those who are closest to them when they are sick.

Our study did not show any association of awareness of medical therapy as acute stroke treatment with age, gender, or education. This contrasts some studies that showed association with young age [15, 18], high education [15, 20], and male gender [18], even though some questions were about the treatment of stroke not specifying acute stroke treatment and were open-ended. The differences can also be explained by differences in the study population, the definition of young age, and the question in our study did not specify the type or name of the medication.

We found that lower awareness of stroke prevention was significantly associated with female gender and being married/cohabiting, which contrasts an Indian study that did not show any association [22]. Our study did not demonstrate any associations with age, which also contrasts the afore-mentioned Ethiopian and Indian studies [21, 22]. Neither did we find any association between awareness and education. This resonates with the Indian study [22] but not with a Chinese study that showed an association with education [24]. Our study showed a significant association of lower awareness of stroke prevention with a healthy diet which contrasts the Indian study that did not show any association with diet [22]. The differences can be explained by differences in the study population (age, region, time of study, type of patients), the way awareness was assessed i.e., based on either a single question or multiple questions, and the nature of questions (either closed- or open-ended).

### Strength and limitations

Our study adds to the sparse literature worldwide on awareness of stroke prevention and acute treatment among outpatients and the public concurrently. This is the first study to access awareness of stroke treatment and prevention after stratifying for age and in addition to sources of information. All information from the questionnaires was collected through standardized face-to-face interviews. In our study assessment of awareness of stroke prevention and acute treatment was performed through closed-ended questions, while awareness of means of stroke risk factors’ prevention was assessed with both open- and closed-ended questions even though closed-ended questions might overestimate the real level of awareness, as revealed by one previous study [33].

There are some limitations to this study. First, not all means of stroke/ risk factors’ prevention were included in this study and should not be weighted equally because some are easily identifiable and more common than others. Second, some subgroups had small samples, therefore, reducing statistical power to calculate differences. Third, self-reported factors/characteristics are prone to bias. Fourth, for acute stroke treatment we did not specify the type of medication or should have given more options for types of medications. Fifth, we could not cover all areas of stroke prevention and treatment as it is a broad topic e.g., thrombectomy, rehabilitation, etc. Lastly, there may be differences in sociodemographic and stroke risk factors between responders and non-responders that we are unable to account for. Despite all these limitations, a reasonably high response rate of 94% was attained and therefore these results represent current knowledge of both outpatients and the public in greater Gaborone.

## Conclusion

Despite adequate awareness of stroke prevention among respondents and adequate awareness of acute stroke treatment among the public for closed-ended questions, there are still gaps in awareness that need to be addressed among various subgroups and even after adjusting for age. The results call health policymakers and other stakeholders for urgent awareness campaigns targeting all these subgroups associated with low awareness as it might help increase the rate of thrombolysis and /thrombectomy, and reduce risk factors through lifestyle interventions and medical adherence hence reduce stroke mortality and morbidity in Sub-Saharan Africa. To increase the numbers reaching time dependent therapy the population needs to know stroke symptoms, i.e. FAST, as awareness of rehabilitation and lifestyle will probably not have magnitude effect on the number available for reperfusion therapy but can have on recovery of poststroke.

## Supplementary Information

Additional file 1.

Additional file 2.

Additional file 3.

Additional file 4.

## Data Availability

The datasets used and analysed during the current study are available by request
