# Supplementary material for: Age and sources of information variations and similarities on awareness of treatment and prevention of stroke among public and outpatients in Sub-Saharan Africa: a cross-sectional questionnaire study in Botswana
Source: BMC Public Health. 2025 Feb 24;25:742. doi: 10.1186/s12889-025-21900-7 (PMC11849147; doi:10.1186/s12889-025-21900-7)
Supplement: Supplementary file 1 — Additional file 1. [file 12889_2025_21900_MOESM1_ESM.docx]

| **eFigure 1. Awareness of acute stroke treatment and prevention study** |
| --- |

**Q1. Patient sociodemographic factors**

| **Gender** | \| **M** \| **F** \| \| --- \| --- \| | **Medical insurance** | \| **Yes** \| **No** \| \| --- \| --- \| |
| --- | --- | --- | --- | --- | --- | --- | --- |
| **Age (years)** |  | **Residing/working together** | \| **Yes** \| **No** \| \| --- \| --- \| |
| **Education** | \| **Primary** \| **Secondary** \| \| --- \| --- \| \| **Tertiary** \| **None** \| | **Marital status** | \| **Married** \| **Single** \| \| --- \| --- \| \| **Cohabiting** \| **Widowed/divorced** \| |
| **Location** | \| **Scot LH** \| **Ram V** \| \| --- \| --- \| \| **Ram RC** \| **Gabz** \| \| **Mosh C** \| **Mosh V** \| \| **Sbrana** \| **Puth C** \| \| **Nko C** \|  \| |  |  |

**Scot LH=Scottish Livingstone Hospital Ram RC=Ramotswa Railway Station clinic**

**Mosh C=Moshupa Clinic Mosh V =Moshupa village Gabz= Gaborone city**

**Sbrana=Sbrana Psychiatric Referral Hospital Nko C=Nkoyaphiri clinic**

**Puth C=Phuthadikobo clinic Ram V=Ramotswa village**

| **Q2. Stroke treatment: Cross correct answer** | | | | | | |
| --- | --- | --- | --- | --- | --- | --- |
| a). Is stroke treatable? | No | | Yes | | No idea | |
|  |  | |  | |  | |
| b). Do you know any ways that treat acute stroke? | Rehabilitation | Lifestyle activities | | Medicine/ medical therapy | | No idea |

| **Q3. Stroke prevention: Cross correct answer** | | | | |
| --- | --- | --- | --- | --- |
| a). Is stroke preventable? | | No | Yes | No idea |
| b). Does reducing/treating stroke risk factors reduce the likelihood of stroke? | | No | Yes | No idea |
| c). Do you know any ways that can reduce stroke risk factors (*open-ended question*)? *Respondents should name answers* |  |  |  |  |
| d). Do you know any ways that can reduce stroke risk factors (*closed-ended questions, mention answers to respondents*)? | No idea | None | Lifestyle | Medicine/ medication |

**Q4a. Self-reporting (or medical reports). Do you have any stroke risk factors?**

|  | **Medical records** | **Self-reported** |  | **Medical records** | **Self-reported** |
| --- | --- | --- | --- | --- | --- |
| **Hypertension** | \| **Yes** \| **No** \| \| --- \| --- \| | \| **Yes** \| **No** \| \| --- \| --- \| | **Sedentary lifestyle** | \| **Yes** \| **No** \| \| --- \| --- \| | \| **Yes** \| **No** \| \| --- \| --- \| |
| **Diabetes** | \| **Yes** \| **No** \| \| --- \| --- \| | \| **Yes** \| **No** \| \| --- \| --- \| | **Smoking** | \| **Yes** \| **No** \| **Ex** \| \| --- \| --- \| --- \| | \| **Yes** \| **No** \| **Ex** \| \| --- \| --- \| --- \| |
| **Dyslipidemia** | \| **Yes** \| **No** \| \| --- \| --- \| | \| **Yes** \| **No** \| \| --- \| --- \| | **Heavy alcohol drinking** | \| **Yes** \| **No** \| **Ex** \| \| --- \| --- \| --- \| | \| **Yes** \| **No** \| **Ex** \| \| --- \| --- \| --- \| |
| **Heart diseases** | \| **Yes** \| **No** \| \| --- \| --- \| |  | **Previous stroke** | \| **Yes** \| **No** \| \| --- \| --- \| | \| **Yes** \| **No** \| \| --- \| --- \| |
| **Family history of stroke** | \| **Yes** \| **No** \| \| --- \| --- \| | \| **Yes** \| **No** \| \| --- \| --- \| | **Family history of both stroke and heart diseases** | \| **Yes** \| **No** \| \| --- \| --- \| | \| **Yes** \| **No** \| \| --- \| --- \| |
| **Family history of heart diseases** | \| **Yes** \| **No** \| \| --- \| --- \| | \| **Yes** \| **No** \| \| --- \| --- \| | **Obesity** | \| **Yes** \| **No** \| \| --- \| --- \| | \| **Yes** \| **No** \| \| --- \| --- \| |
| **HIV/AIDS**  **Other risk factors:**  **1……………**  **2……………**  **3……………** | \| **Yes** \| **No** \| \| --- \| --- \|  \| **Yes** \| **No** \| \| --- \| --- \| \| **Yes** \| **No** \| \| **Yes** \| **No** \| | \| **Yes** \| **No** \| \| --- \| --- \|  \| **Yes** \| **No** \| \| --- \| --- \| \| **Yes** \| **No** \| \| **Yes** \| **No** \| | **Psychiatric disease**  **In case yes, which ones do you have?**  **…………………**  **………………….** | \| **Yes** \| **No** \| \| --- \| --- \|  \| **Yes** \| **No** \| \| --- \| --- \| \| **Yes** \| **No** \| | \| **Yes** \| **No** \| \| --- \| --- \|  \| **Yes** \| **No** \| \| --- \| --- \| \| **Yes** \| **No** \| |

| **What do you think of your weight?** | \| **No idea** \| **Normal** \| **Obese** \| \| --- \| --- \| --- \| | \| **Underweight** \| **Overweight** \| \| --- \| --- \| |
| --- | --- | --- | --- | --- | --- | --- | --- |
| **Do you think you eat healthy?** | \| **Yes** \| **No** \| **No idea** \| \| --- \| --- \| --- \| | **Height 1 (cm):**  **Height 2 (cm):**  **Weight (kg):** |

**Q4b. Physical activities**

| **Do you do any physical activity?** | \| **Yes** \| **No** \| \| --- \| --- \| |
| --- | --- | --- | --- |
| **In case yes, what type of physical activities do you do?** | \| **1.** \| **2.** \| \| --- \| --- \| \| **3.** \| **4.** \| |
| **How many times a day?** | \| **1.** \| **2.** \| \| --- \| --- \| \| **3.** \| **4.** \| |
| **How many minutes in a day?** | \| **1.** \| **2.** \| \| --- \| --- \| \| **3.** \| **4.** \| |
| **How many times in a week?** | \| **1.** \| **2.** \| \| --- \| --- \| \| **3.** \| **4.** \| |
| **How will you grade the intensity of your physical activity?** | \| **1. Inactive** \| **2. Low** \| \| --- \| --- \| \| **3. Moderate** \| **4. High** \| \| **5. No idea** \|  \| |

**Q5. Sources of stroke information**

| **How did you get to know about stroke? (*closed-ended question*). Multiple answers**  ***Mention them to respondents*** | |
| --- | --- |
|  | Cross right answers |
| **TV/ radio** |  |
| **Newspaper/ magazines** |  |
| **Family/ friends** |  |
| **Doctors/ nurses** |  |
| **Social media (internet, Whatsapp, facebook, Instagram, etc)** |  |
| **Others (school, experience, patients)** |  |
