# Supplementary material for: Age and sources of information variations and similarities on awareness of treatment and prevention of stroke among public and outpatients in Sub-Saharan Africa: a cross-sectional questionnaire study in Botswana
Source: BMC Public Health. 2025 Feb 24;25:742. doi: 10.1186/s12889-025-21900-7 (PMC11849147; doi:10.1186/s12889-025-21900-7)
Supplement: Supplementary file 2 — Additional file 2. [file 12889_2025_21900_MOESM2_ESM.docx]

|  |  |  |  |  |  |  |
| --- | --- | --- | --- | --- | --- | --- |
| **eTable 1. Awareness of acute treatment and prevention of stroke stratified by age** | | | | | |  |
|  |  |  |  |  |  |  |
| **Respondents' type** |  |  | **Aware of medical therapy as an acute treatment option** | | | |
| **Age (years)** | **Aware of the possibility of acute stroke treatment** | All | Yes | No |  |  |
|  |  | n | n | n | *p* | **OR** |
|  |  |  |  |  |  |  |
| **Total** |  |  |  |  |  |  |
| All ages | Yes | 2487 | 1861 | 626 | <0.001 | 1872.9* |
|  | No | 315 | 0 | 315 |  |  |
| 18-34 | Yes | 1323 | 1016 | 307 | <0.001 | 1178.2* |
|  | No | 178 | 0 | 178 |  |  |
| 35-49 | Yes | 757 | 549 | 208 | <0.001 | 448.7* |
|  | No | 85 | 0 | 85 |  |  |
| >50 | Yes | 407 | 296 | 111 | <0.001 | 277.3* |
|  | No | 52 | 0 | 52 |  |  |
|  |  |  |  |  |  |  |
| **Public** |  |  |  |  |  |  |
| All ages | Yes | 1766 | 1517 | 249 | <0.001 | 2948.7* |
|  | No | 242() | 0 | 242 |  |  |
| 18-34 | Yes | 978 | 848 | 130 | <0.001 | 1826.5* |
|  | No | 140 | 0 | 140 |  |  |
| 35-49 | Yes | 525 | 443 | 82 | <0.001 | 659.1* |
|  | No | 61 | 0 | 61 |  |  |
| >50 | Yes | 263 | 226 | 37 | <0.001 | 500.9* |
|  | No | 41 | 0 | 41 |  |  |
|  |  |  |  |  |  |  |
| **Outpatients** |  |  |  |  |  |  |
| All ages | Yes | 722 | 344 | 377 | <0.001 | 133.2* |
|  | No | 73 | 0 | 73 |  |  |
| 18-34 | Yes | 345 | 168 | 177 | <0.001 | 72.1* |
|  | No | 38 | 0 | 38 |  |  |
| 35-49 | Yes | 232 | 106 | 126 | 0.002 | 40.4* |
|  | No | 24 | 0 | 24 |  |  |
| >50 | Yes | 144 | 70 | 74 | 0.010 | 20.8* |
|  | No | 11 | 0 | 11 |  |  |
|  |  |  |  |  |  |  |
|  | **Aware of the possibility of stroke prevention** |  | **Aware that reducing or treating stroke risk factors prevents stroke** | | |  |
| **Total** |  |  |  |  |  |  |
| All ages | Yes | 2084 | 2024 | 60 | <0.001 | 21.2 |
|  | No | 718 | 441 | 277 |  |  |
| 18-34 | Yes | 1124 | 1094 | 30 | <0.001 | 20.8 |
|  | No | 377 | 240 | 137 |  |  |
| 35-49 | Yes | 619 | 602 | 17 | <0.001 | 23.1 |
|  | No | 223 | 135 | 88 |  |  |
| >50 | Yes | 341 | 328 | 13 | <0.001 | 19.9 |
|  | No | 118 | 66 | 52 |  |  |
|  |  |  |  |  |  |  |
| **Public** |  |  |  |  |  |  |
| All ages | Yes | 1441 | 1393 | 48 | <0.001 | 21.9 |
|  | No | 572 | 326 | 246 |  |  |
| 18-34 | Yes | 819 | 794 | 25 | <0.001 | 21.9 |
|  | No | 299 | 177 | 122 |  |  |
| 35-49 | Yes | 411 | 397 | 14 | <0.001 | 22.3 |
|  | No | 175 | 98 | 77 |  |  |
| >50 | Yes | 207 | 198 | 9 | <0.001 | 20.7 |
|  | No | 97 | 50 | 47 |  |  |
|  |  |  |  |  |  |  |
| **Outpatients** |  |  |  |  |  |  |
| All ages | Yes | 648 | 636 | 12 | <0.001 | 14.2 |
|  | No | 147 | 116 | 31 |  |  |
| 18-34 | Yes | 305 | 300 | 5 | <0.001 | 14.3 |
|  | No | 78 | 63 | 15 |  |  |
| 35-49 | Yes | 208 | 205 | 3 | <0.001 | 20.3# |
|  | No | 48 | 37 | 11 |  |  |
| >50 | Yes | 134 | 130 | 4 | 0.003 | 10.2# |
|  | No | 21 | 16 | 5 |  |  |
|  |  |  |  |  |  |  |
|  | **Aware of the possibility of stroke prevention** |  | **Able to spontaneously recall at least one way of reducing or treating stroke risk factors** | | | |
| **Total** |  |  |  |  |  |  |
| All ages | Yes | 2084 | 627 | 1457 | <0.001 | 0.6 |
|  | No | 718 | 305 | 413 |  |  |
| 18-34 | Yes | 1124 | 359 | 765 | <0.001 | 0.6 |
|  | No | 377 | 172 | 205 |  |  |
| 35-49 | Yes | 619 | 195 | 424 | 0.003 | 0.6 |
|  | No | 223 | 93 | 130 |  |  |
| >50 | Yes | 341 | 73 | 268 | 0.004 | 0.5 |
|  | No | 118 | 40 | 78 |  |  |
| **Public** |  |  |  |  |  |  |
| All ages | Yes | 1437 | 456 | 981 | <0.001 | 0.7 |
|  | No | 571 | 230 | 341 |  |  |
| 18-34 | Yes | 819 | 260 | 559 | <0.001 | 0.5 |
|  | No | 299 | 138 | 161 |  |  |
| 35-49 | Yes | 411 | 148 | 263 | 0.45 |  |
|  | No | 175 | 64 | 111 |  |  |
| >50 | Yes | 207 | 48 | 159 | 0.14 |  |
|  | No | 97 | 28 | 69 |  |  |
| **Outpatients** |  |  |  |  |  |  |
| All ages | Yes | 647 | 171 | 476 | <0.001 | 0.3 |
|  | No | 147 | 75 | 72 |  |  |
| 18-34 | Yes | 305 | 99 | 206 | 0.03 | 0.6 |
|  | No | 78 | 34 | 44 |  |  |
| 35-49 | Yes | 208 | 47 | 161 | <0.001 | 0.2 |
|  | No | 48 | 29 | 19 |  |  |
| >50 | Yes | 134 | 25 | 109 | <0.001 | 0.2 |
|  | No | 21 | 12 | 9 |  |  |
|  |  |  |  |  |  |  |
|  | **Aware of the possibility of stroke prevention** |  | **Able to recognize at least one way of reducing or treating stroke risk factors** | | | |
| **Total** |  |  |  |  |  |  |
| All ages | Yes | 2084 | 2036 | 48 | <0.001 | 7.8 |
|  | No | 718 | 606 | 112 |  |  |
| 18-34 | Yes | 1124 | 1098 | 26 | <0.001 | 8.2 |
|  | No | 377 | 316 | 61 |  |  |
| 35-49 | Yes | 619 | 611 | 8 | <0.001 | 9.2 |
|  | No | 223 | 199 | 24 |  |  |
| >50 | Yes | 341 | 327 | 14 | <0.001 | 6.9 |
|  | No | 118 | 91 | 27 |  |  |
| **Public** |  |  |  |  |  |  |
| All ages | Yes | 1437 | 1397 | 40 | <0.001 | 6.6 |
|  | No | 571 | 480 | 91 |  |  |
| 18-34 | Yes | 819 | 795 | 24 | <0.001 | 5.9 |
|  | No | 299 | 254 | 45 |  |  |
| 35-49 | Yes | 411 | 405 | 6 | <0.001 | 9.7 |
|  | No | 175 | 153 | 22 |  |  |
| >50 | Yes | 207 | 197 | 10 | <0.001 | 6.5 |
|  | No | 97 | 73 | 24 |  |  |
|  |  |  |  |  |  |  |
| **Outpatients** |  |  |  |  |  |  |
| All ages | Yes | 647 | 639 | 8 | <0.001 | 13.3 |
|  | No | 147 | 126 | 21 |  |  |
| 18-34 | Yes | 305 | 303 | 2 | <0.001 | 39.1# |
|  | No | 78 | 62 | 16 |  |  |
| 35-49 | Yes | 208 | 206 | 2 | 0.16 |  |
|  | No | 48 | 46 | 2 |  |  |
| >50 | Yes | 134 | 130 | 4 | 0.05 |  |
|  | No | 21 | 18 | 3 |  |  |
|  |  |  |  |  |  |  |
|  |  |  |  |  |  |  |
| OR: odds ratio, *: Haldane-Anscombe correction, #: used Fisher exact | | | | |  |  |
|  |  |  |  |  |  |  |
|  |  |  |  |  |  |  |
