# Supplementary material for: Age and sources of information variations and similarities on awareness of treatment and prevention of stroke among public and outpatients in Sub-Saharan Africa: a cross-sectional questionnaire study in Botswana
Source: BMC Public Health. 2025 Feb 24;25:742. doi: 10.1186/s12889-025-21900-7 (PMC11849147; doi:10.1186/s12889-025-21900-7)
Supplement: Supplementary file 3 — Additional file 3. [file 12889_2025_21900_MOESM3_ESM.docx]

|  |  |  |  |  |  |  |
| --- | --- | --- | --- | --- | --- | --- |
| **eTable 2. Awareness of medical therapy as acute treatment of stroke and of stroke prevention by sources of stroke information stratified by age** | | | | | | |
|  |  |  |  |  |  |  |
|  |  | Total |  |  |  |  |
| Age (years) |  | n | No. aware | No. unaware | *p* | OR |
|  |  |  |  |  |  |  |
| **Awareness of medical therapy as acute treatment** | | | | |  |  |
| **PUBLIC** |  |  |  |  |  |  |
| **Family or friends** | |  |  |  |  |  |
| All | Yes | 1216 | 938 | 278 | 0.040 | 1.2 |
|  | No | 792 | 579 | 213 |  |  |
| 18-34 | Yes | 651 | 497 | 154 | 0.648 |  |
|  | No | 467 | 351 | 116 |  |  |
| 35-49 | Yes | 370 | 292 | 78 | 0.015 | 1.6 |
|  | No | 216 | 151 | 65 |  |  |
| >50 | Yes | 195 | 149 | 46 | 0.270 |  |
|  | No | 109 | 77 | 32 |  |  |
| **Tv or radio** |  |  |  |  |  |  |
| All | Yes | 1152 | 950 | 202 | <0.001 | 2.4 |
|  | No | 856 | 567 | 289 |  |  |
| 18-34 | Yes | 650 | 543 | 107 | <0.001 | 2.7 |
|  | No | 468 | 305 | 163 |  |  |
| 35-49 | Yes | 337 | 276 | 61 | <0.001 | 2.2 |
|  | No | 249 | 167 | 82 |  |  |
| >50 | Yes | 165 | 131 | 34 | 0.029 | 1.8 |
|  | No | 139 | 95 | 44 |  |  |
| **Newspaper or magazine** | |  |  |  |  |  |
| All | Yes | 799 | 675 | 124 | <0.001 | 2.4 |
|  | No | 1209 | 842 | 367 |  |  |
| 18-34 | Yes | 463 | 407 | 56 | <0.001 | 3.5 |
|  | No | 655 | 441 | 214 |  |  |
| 35-49 | Yes | 256 | 209 | 47 | 0.003 | 1.8 |
|  | No | 330 | 234 | 96 |  |  |
| >50 | Yes | 80 | 59 | 21 | 0.888 |  |
|  | No | 224 | 167 | 57 |  |  |
| **Social Media (Internet, Facebook, WhatsApp)** | | | |  |  |  |
| All | Yes | 542 | 456 | 86 | <0.001 | 2.0 |
|  | No | 1466 | 1061 | 405 |  |  |
| 18-34 | Yes | 312 | 269 | 43 | <0.001 | 2.5 |
|  | No | 284 | 579 | 227 |  |  |
| 35-49 | Yes | 194 | 159 | 35 | 0.012 | 1.7 |
|  | No | 392 | 284 | 108 |  |  |
| >50 | Yes | 36 | 28 | 8 | 0.616 |  |
|  | No | 268 | 198 | 70 |  |  |
| **Doctor or nurse** | |  |  |  |  |  |
| All | Yes | 777 | 674 | 103 | <0.001 | 3.0 |
|  | No | 1231 | 843 | 388 |  |  |
| 18-34 | Yes | 438 | 399 | 39 | <0.001 | 5.3 |
|  | No | 680 | 449 | 231 |  |  |
| 35-49 | Yes | 245 | 201 | 44 | 0.002 | 1.9 |
|  | No | 341 | 242 | 99 |  |  |
| >50 | Yes | 94 | 74 | 20 | 0.243 |  |
|  | No | 210 | 152 | 58 |  |  |
| **Others (school, patients, experience)** | | | |  |  |  |
| All | Yes | 330 | 185 | 145 | <0.001 | 0.3 |
|  | No | 1678 | 1332 | 346 |  |  |
| 18-34 | Yes | 208 | 122 | 86 | <0.001 | 0.4 |
|  | No | 910 | 726 | 184 |  |  |
| 35-49 | Yes | 78 | 42 | 36 | <0.001 | 0.3 |
|  | No | 508 | 401 | 107 |  |  |
| >50 | Yes | 44 | 21 | 23 | <0.001 | 0.2 |
|  | No | 260 | 205 | 55 |  |  |
|  |  |  |  |  |  |  |
| **OUTPATIENTS** | |  |  |  |  |  |
| **Family or friends** | |  |  |  |  |  |
| All | Yes | 496 | 238 | 258 | <0.001 | 1.7 |
|  | No | 298 | 106 | 192 |  |  |
| 18-34 | Yes | 241 | 121 | 120 | 0.001 | 2.0 |
|  | No | 142 | 47 | 95 |  |  |
| 35-49 | Yes | 147 | 64 | 83 | 0.422 |  |
|  | No | 109 | 42 | 67 |  |  |
| >50 | Yes | 108 | 53 | 55 | 0.140 |  |
|  | No | 47 | 17 | 30 |  |  |
| **Tv or radio** |  |  |  |  |  |  |
| All | Yes | 506 | 190 | 316 | <0.001 | 0.5 |
|  | No | 288 | 154 | 134 |  |  |
| 18-34 | Yes | 228 | 84 | 144 | <0.001 | 0.5 |
|  | No | 155 | 84 | 71 |  |  |
| 35-49 | Yes | 179 | 66 | 113 | 0.026 | 0.5 |
|  | No | 77 | 40 | 37 |  |  |
| >50 | Yes | 99 | 40 | 59 | 0.115 |  |
|  | No | 56 | 30 | 26 |  |  |
| **Newspaper or magazine** | |  |  |  |  |  |
| All | Yes | 432 | 162 | 270 | <0.001 | 0.6 |
|  | No | 362 | 182 | 180 |  |  |
| 18-34 | Yes | 204 | 81 | 123 | 0.080 |  |
|  | No | 179 | 87 | 92 |  |  |
| 35-49 | Yes | 154 | 52 | 102 | 0.003 | 0.5 |
|  | No | 102 | 54 | 48 |  |  |
| >50 | Yes | 74 | 29 | 45 | 0.154 |  |
|  | No | 81 | 41 | 40 |  |  |
| **Social Media (Internet, Facebook, WhatsApp)** | | | |  |  |  |
| All | Yes | 186 | 59 | 127 | <0.001 | 0.5 |
|  | No | 608 | 285 | 323 |  |  |
| 18-34 | Yes | 109 | 40 | 69 | 0.076 |  |
|  | No | 274 | 128 | 146 |  |  |
| 35-49 | Yes | 57 | 14 | 43 | 0.004 | 0.4 |
|  | No | 199 | 92 | 107 |  |  |
| >50 | Yes | 20 | 5 | 15 | 0.058* |  |
|  | No | 135 | 65 | 70 |  |  |
| **Doctor or nurse** | |  |  |  |  |  |
| All | Yes | 290 | 112 | 178 | 0.043 | 0.7 |
|  | No | 504 | 232 | 272 |  |  |
| 18-34 | Yes | 139 | 58 | 81 | 0.525 |  |
|  | No | 244 | 110 | 134 |  |  |
| 35-49 | Yes | 97 | 32 | 65 | 0.034 | 0.6 |
|  | No | 159 | 74 | 85 |  |  |
| >50 | Yes | 54 | 22 | 32 | 0.419 |  |
|  | No | 101 | 48 | 53 |  |  |
| **Others (school, patients, experience)** | | | |  |  |  |
| All | Yes | 129 | 74 | 55 | <0.001 | 2.0 |
|  | No | 665 | 270 | 395 |  |  |
| 18-34 | Yes | 83 | 48 | 35 | 0.004 | 2.1 |
|  | No | 300 | 120 | 180 |  |  |
| 35-49 | Yes | 22 | 14 | 8 | 0.032 | 2.7 |
|  | No | 234 | 92 | 142 |  |  |
| >50 | Yes | 24 | 12 | 12 | 0.605 |  |
|  | No | 131 | 58 | 73 |  |  |
|  |  |  |  |  |  |  |
| **Awarenes of stroke prevention** | | |  |  |  |  |
| **PUBLIC** |  |  |  |  |  |  |
| **Family or friends** | |  |  |  |  |  |
| All | Yes | 1216 | 857 | 359 | 0.181 |  |
|  | No | 792 | 580 | 212 |  |  |
| 18-34 | Yes | 651 | 476 | 175 | 0.902 |  |
|  | No | 467 | 343 | 124 |  |  |
| 35-49 | Yes | 370 | 250 | 120 | 0.076 |  |
|  | No | 216 | 161 | 55 |  |  |
| >50 | Yes | 195 | 131 | 64 | 0.648 |  |
|  | No | 109 | 76 | 33 |  |  |
| **Tv or radio** |  |  |  |  |  |  |
| All | Yes | 1152 | 896 | 256 | <0.001 | 2.0 |
|  | No | 856 | 541 | 315 |  |  |
| 18-34 | Yes | 650 | 526 | 124 | <0.001 | 2.5 |
|  | No | 468 | 293 | 175 |  |  |
| 35-49 | Yes | 337 | 248 | 89 | 0.034 | 1.5 |
|  | No | 249 | 163 | 86 |  |  |
| >50 | Yes | 165 | 122 | 43 | 0.018 | 1.8 |
|  | No | 139 | 85 | 54 |  |  |
| **Newspaper or magazine** | |  |  |  |  |  |
| All | Yes | 799 | 645 | 154 | <0.001 | 2.2 |
|  | No | 1209 | 792 | 417 |  |  |
| 18-34 | Yes | 463 | 393 | 70 | <0.001 | 3.0 |
|  | No | 655 | 426 | 229 |  |  |
| 35-49 | Yes | 256 | 187 | 69 | 0.176 |  |
|  | No | 330 | 224 | 106 |  |  |
| >50 | Yes | 80 | 65 | 15 | 0.004 | 2.5 |
|  | No | 224 | 142 | 82 |  |  |
| **Social Media (Internet, Facebook, WhatsApp)** | | | |  |  |  |
| All | Yes | 542 | 420 | 122 | 0.004 | 1.5 |
|  | No | 1466 | 1017 | 449 |  |  |
| 18-34 | Yes | 312 | 256 | 56 | <0.001 | 2.0 |
|  | No | 806 | 563 | 243 |  |  |
| 35-49 | Yes | 194 | 138 | 56 | 0.711 |  |
|  | No | 392 | 273 | 119 |  |  |
| >50 | Yes | 36 | 26 | 10 | 0.572 |  |
|  | No | 268 | 181 | 87 |  |  |
| **Doctor or nurse** | |  |  |  |  |  |
| All | Yes | 777 | 608 | 169 | <0.001 | 1.7 |
|  | No | 1231 | 829 | 402 |  |  |
| 18-34 | Yes | 438 | 367 | 71 | <0.001 | 2.6 |
|  | No | 680 | 452 | 228 |  |  |
| 35-49 | Yes | 245 | 173 | 72 | 0.831 |  |
|  | No | 341 | 238 | 103 |  |  |
| >50 | Yes | 94 | 68 | 26 | 0.289 |  |
|  | No | 210 | 139 | 71 |  |  |
| **Others (school, patients, experience)** | | | |  |  |  |
| All | Yes | 330 | 208 | 122 | <0.001 | 0.6 |
|  | No | 1678 | 1229 | 449 |  |  |
| 18-34 | Yes | 208 | 127 | 81 | <0.001 | 0.5 |
|  | No | 910 | 692 | 218 |  |  |
| 35-49 | Yes | 78 | 58 | 20 | 0.382 |  |
|  | No | 508 | 353 | 155 |  |  |
| >50 | Yes | 44 | 23 | 21 | 0.017 | 0.5 |
|  | No | 260 | 184 | 76 |  |  |
|  |  |  |  |  |  |  |
| **OUTPATIENTS** | |  |  |  |  |  |
| **Family or friends** | |  |  |  |  |  |
| All | Yes | 496 | 416 | 80 | 0.026 | 1.5 |
|  | No | 298 | 231 | 67 |  |  |
| 18-34 | Yes | 241 | 200 | 41 | 0.035 | 1.7 |
|  | No | 142 | 105 | 37 |  |  |
| 35-49 | Yes | 147 | 122 | 25 | 0.407 |  |
|  | No | 109 | 86 | 23 |  |  |
| >50 | Yes | 108 | 94 | 14 | 0.747 |  |
|  | No | 47 | 40 | 7 |  |  |
| **Tv or radio** |  |  |  |  |  |  |
| All | Yes | 506 | 435 | 71 | <0.001 | 2.2 |
|  | No | 288 | 212 | 76 |  |  |
| 18-34 | Yes | 228 | 189 | 39 | 0.056 |  |
|  | No | 155 | 116 | 39 |  |  |
| 35-49 | Yes | 179 | 155 | 24 | 0.001 | 2.9 |
|  | No | 77 | 53 | 24 |  |  |
| >50 | Yes | 99 | 91 | 8 | 0.011 | 3.4 |
|  | No | 56 | 43 | 13 |  |  |
| **Newspaper or magazine** | |  |  |  |  |  |
| All | Yes | 432 | 388 | 44 | <0.001 | 3.5 |
|  | No | 362 | 259 | 103 |  |  |
| 18-34 | Yes | 204 | 176 | 28 | <0.001 | 2.4 |
|  | No | 179 | 129 | 50 |  |  |
| 35-49 | Yes | 154 | 142 | 12 | <0.001 | 6.5 |
|  | No | 102 | 66 | 36 |  |  |
| >50 | Yes | 74 | 70 | 4 | 0.008 | 4.6 |
|  | No | 81 | 64 | 17 |  |  |
| **Social Media (Internet, Facebook, WhatsApp)** | | | |  |  |  |
| All | Yes | 186 | 160 | 26 | 0.070 |  |
|  | No | 608 | 487 | 121 |  |  |
| 18-34 | Yes | 109 | 93 | 16 | 0.084 |  |
|  | No | 274 | 212 | 62 |  |  |
| 35-49 | Yes | 57 | 49 | 8 | 0.304 |  |
|  | No | 199 | 159 | 40 |  |  |
| >50 | Yes | 20 | 18 | 2 | 0.999* |  |
|  | No | 135 | 116 | 19 |  |  |
| **Doctor or nurse** | |  |  |  |  |  |
| All | Yes | 290 | 246 | 44 | 0.067 |  |
|  | No | 504 | 401 | 103 |  |  |
| 18-34 | Yes | 139 | 116 | 23 | 0.163 |  |
|  | No | 244 | 189 | 55 |  |  |
| 35-49 | Yes | 97 | 84 | 13 | 0.090 |  |
|  | No | 159 | 124 | 35 |  |  |
| >50 | Yes | 54 | 46 | 8 | 0.736 |  |
|  | No | 101 | 88 | 13 |  |  |
| **Others (school, patients, experience)** | | | |  |  |  |
| All | Yes | 129 | 99 | 30 | 0.131 |  |
|  | No | 665 | 548 | 117 |  |  |
| 18-34 | Yes | 83 | 68 | 15 | 0.558 |  |
|  | No | 300 | 237 | 63 |  |  |
| 35-49 | Yes | 22 | 17 | 5 | 0.575* |  |
|  | No | 234 | 191 | 43 |  |  |
| >50 | Yes | 24 | 14 | 10 | <0.001 | 0.1 |
|  | No | 131 | 120 | 11 |  |  |
|  |  |  |  |  |  |  |
| OR:odds ratio, *:used Fisher exact | | |  |  |  |  |
|  |  |  |  |  |  |  |
|  |  |  |  |  |  |  |
